# Four Patients with COVID-19 and Tuberculosis, Singapore, April–May 2020

**DOI:** 10.3201/eid2611.202752

**Published:** 2020-11

**Authors:** Sai Meng Tham, Wei Yang Lim, Chun Kiat Lee, Jerold Loh, Arthi Premkumar, Benedict Yan, Adrian Kee, Louis Chai, Paul Anantharajah Tambyah, Gabriel Yan

**Affiliations:** National University Health System, Singapore

**Keywords:** 2019 novel coronavirus disease, coronavirus disease, COVID-19, severe acute respiratory syndrome coronavirus 2, SARS-CoV-2, viruses, respiratory infections, zoonoses, tuberculosis, tuberculosis and other mycobacteria, co-infection, socioeconomic, radiography, Singapore

## Abstract

Coronavirus disease (COVID-19) and tuberculosis (TB) developed in 4 foreign workers living in dormitories in Singapore during April–May 2020. Clinical manifestations and atypical radiographic features of COVID-19 led to the diagnosis of TB through positive interferon-gamma release assay and culture results. During the COVID-19 pandemic, TB should not be overlooked.

As the world focuses on the coronavirus disease (COVID-19) pandemic, caution must be taken to not overlook tuberculosis (TB). COVID-19 was first diagnosed in Singapore in January 2020, after cases were imported from Wuhan, China. Subsequent sustained community transmission of the virus followed a wave of imported cases from local residents returning from abroad (*1*). The outbreak in Singapore is being driven by spread within migrant worker dormitories. As of June 28, 2020, Singapore reported 43,459 confirmed cases of COVID-19, of which 41,010 were in dormitory residents (*1*). TB is endemic to Singapore; annual incidence rate is ≈40 cases/100,000 population (*2*), and a large proportion of cases are in nonpermanent residents.

We describe migrant workers in Singapore dormitories who were co-infected with severe acute respiratory syndrome virus 2 (SARS-CoV-2) and *Mycobacterium tuberculosis*. For all 4 patients, TB was diagnosed by positive interferon-gamma release assay (IGRA) (QIAGEN, https://www.qiagen.com); *M. tuberculosis* was isolated from pleural fluid culture from patient 4 only (Table).

**Table:** Epidemiologic and clinical features for 4 patients with coronavirus disease and tuberculosis, Singapore*

Pt no.	Age, y/sex, nationality	Initial signs/symptoms	Radiologic findings	Pleural fluid analysis	Sputum analysis	Microbiological findings	IGRA for TB	Outcome
1†	32/M, India	Fever, productive cough	CXR: right upper zone and left lower zone cavitary lesions; chest CT: irregular opacifications with central cavitation	NA	AFB smear negative; molecular TB analysis negative	Sputum AFB culture negative	+	Symptoms resolved; repeat CXR after starting ATT demonstrated resolution of cavitary lesions at 2 mo of treatment
2	33/M, India	Fever, nonproductive cough; 3-kg weight loss over 1 mo	CXR: right-sided pleural effusion; chest CT: loculated right-sided effusion with adjacent collapse/consolidation	Lymphocytic exudative effusion; ADA 130 U/L; SARS-CoV-2 PCR negative	AFB smear negative; molecular TB analysis negative	Sputum and pleural fluid AFB cultures pending	+	Symptoms resolved with interval improvement of CXR
3†	22/M, India	Fever, nonproductive cough; exertional dyspnea, pleuritic chest pain	CXR: right-sided pleural effusion with adjacent compressive atelectasis	Lymphocytic exudative effusion; ADA 112 U/L; SARS-CoV-2 PCR negative	AFB smear negative; molecular TB analysis negative	Sputum and pleural fluid AFB cultures pending	+	Symptoms resolved with interval improvement of CXR
4	40/M, Bangladesh	Fever, productive cough; reduced effort tolerance	CXR: large left-sided pleural effusion; Chest CT: left-sided pleural effusion, bilateral patchy consolidative changes with ground-glass opacities and interlobular septal thickening	Lymphocytic exudative effusion; ADA 69 U/L; SARS-CoV-2 PCR negative	AFB smear negative; molecular TB analysis negative	Sputum AFB culture negative; pleural fluid AFB culture positive for *Mycobacterium tuberculosis* complex	+	Symptoms resolved with interval improvement of CXR

*ADA, adenosine deaminase; AFB, acid-fast bacilli; ATT, anti-TB therapy; CT, computed tomography image; CXR, plain chest radiograph; IGRA, interferon gamma release assay; NA, not applicable; Pt, patient; SARS-CoV-2, severe acute respiratory syndrome coronavirus 2; TB, tuberculosis; +, positive. †These patients reside in the same dormitory.

Patient 1 was a 32-year-old man from India with a 2-day history of fever and cough. He was positive for SARS-CoV-2 by reverse transcription PCR (RT-PCR) of a nasopharyngeal swab sample. Radiographs showed bilateral cavitary lung lesions (Figure 1, panel A). Sputum samples were smear negative and culture negative for acid-fast bacilli (AFB) and negative by molecular testing for *M. tuberculosis* nucleic acids (Cepheid Xpert MTB/RIF, https://www.cepheid.com). The IGRA for TB result was positive. In consideration of the clinical manifestations and risk factors, anti-TB therapy (ATT) was started, and interval radiographic imaging showed resolution.

Patient 2 was a 33-year-old man from India with an 8-day history of fever and cough and a 1-month history of weight loss (3 kg). He was positive for SARS-CoV-2 by RT-PCR of a nasopharyngeal swab sample. Radiographs showed a right-sided pleural effusion (Figure 1, panel B). Pleural fluid analysis revealed a lymphocytic exudative effusion with an adenosine deaminase (ADA) level of 130 U/L (reference range <40 U/L), but the fluid was negative for SARS-CoV-2 by RT-PCR. Sputum and pleural fluid were smear negative for AFB and *M. tuberculosis* nucleic acid negative by molecular testing; culture results are pending. IGRA was positive for TB, and ATT was started with subsequent clinical improvement.

Patient 3 was a 22-year old man from India with a 10-day history of fever and cough (associated with exertional dyspnea) and pleuritic chest pain. He was positive for SARS-CoV-2 by RT-PCR of a nasopharyngeal swab sample. Radiographs showed a right-sided pleural effusion (Figure 1, panel C). Pleural fluid analysis revealed a lymphocytic exudative effusion with an ADA level of 112 U/L and interleukin-6 (IL-6) level of >1,000 pg/mL, but the fluid was negative for SARS-CoV-2 by RT-PCR. Sputum and pleural fluid were smear negative for AFB and negative for *M. tuberculosis* nucleic acids by molecular testing; culture results are pending. IGRA was positive for TB and ATT was started; symptoms subsequently resolved.

Patient 4 was a 40-year old man from Bangladesh with a 3-day history of fever and cough. He was positive for SARS-CoV-2 by RT-PCR from a nasopharyngeal swab sample. Radiographs showed a left-sided pleural effusion with bilateral consolidation (Figure 1, panel D). Pleural fluid analysis revealed a lymphocytic exudative effusion with an ADA level of 62 U/L and an IL-6 level of >1,000 pg/mL, but the fluid was negative for SARS-CoV-2 by RT-PCR. Sputum and pleural fluid were smear negative for AFB and negative for *M. tuberculosis* nucleic acids by molecular testing, but the IGRA for TB was positive. ATT was started, and pleural fluid cultures were subsequently positive for *M. tuberculosis*.

All 4 patients were workers who resided in dormitories and had COVID-19 but atypical radiographic features; typical radiographic features for COVID-19 patients include ground-glass opacities, multifocal patchy consolidation, and peripheral interstitial changes (*3*). Despite confirmed diagnoses of COVID-19, the 4 patients’ pulmonary radiologic findings were more consistent with those for TB, highlighting the value of considering other pulmonary pathologic conditions for patients with COVID-19.

Risk factors for TB include low socioeconomic status and overcrowded living conditions (*4*). Of note, patients 1 and 3 resided in the same dormitory. Migrant worker dormitories are often inadequately ventilated and crowded, resulting in residents being more susceptible to infectious diseases, including dengue, Zika, and varicella (*5*,*6*). The same working and living conditions have served as a catalyst for the rapid transmission of SARS-CoV-2, and potentially TB, in this population. Improving screening processes and living conditions and implementing routine vaccination strategies for this population may prevent future infectious disease outbreaks.

As the COVID-19 pandemic continues, care for patients with TB may be compromised as additional strains are placed on essential services. The 4 cases we report highlight a serious public health issue. Precautionary measures must be undertaken to be vigilant of an epidemic within the ongoing pandemic—TB. To ensure that care is not compromised, clinicians treating these at-risk populations should be aware of possible co-infection with *M. tuberculosis* and SARS-CoV-2 in patients with atypical radiographic features of COVID-19.

**Figure:**
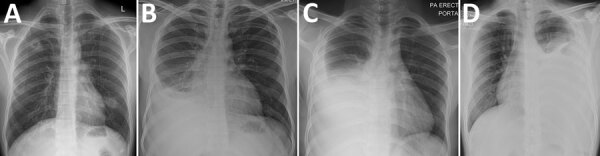
Plain chest radiographs of 4 patients with severe acute respiratory syndrome coronavirus 2 and *Mycobacterium tuberculosis* co-infection, Singapore. A) Patient 1, showing bilateral cavitary lesions; B) patient 2, showing a large right-sided loculated pleural effusion and adjacent consolidation; C) patient 3, showing a large right-sided pleural effusion with adjacent compressive atelectasis; D) patient 4, showing a large left-sided pleural effusion with adjacent consolidation.
